# Exacerbated lung inflammation in offspring with high maternal antibody levels following secondary RSV exposure

**DOI:** 10.3389/fimmu.2024.1377374

**Published:** 2024-04-30

**Authors:** Jinhua Ma, Ting Gong, Tingting Luo, Shuanglian Li, Li Zhong, Xin Zhao, Chenghao Mei, Huaqin Bu, Zhenxing Jia, Xiaohu Kuang, Xiaoli Wang, Zhou Fu, Daiyin Tian

**Affiliations:** ^1^ Department of Respiratory Medicine Children’s Hospital of Chongqing Medical University, National Clinical Research Center for Child Health and Disorders, Ministry of Education Key Laboratory of Child Development and Disorders, Chongqing Key Laboratory of Child Rare Diseases in Infection and Immunity, Chongqing, China; ^2^ Department of mAbs Discovery, Zhuhai Trinomab Pharmaceutical Co., Ltd, Zhuhai, China; ^3^ Department of Respiratory Medicine, Yibin Hospital Affiliated to Children’s Hospital of Chongqing Medical University, Yibin, China

**Keywords:** respiratory syncytial virus, maternal immunization, secondary RSV exposure, mucosal immunity, type 2 inflammation

## Abstract

Respiratory syncytial virus (RSV) is the primary cause of bronchiolitis-related hospitalizations among children under 5 years of age, with reinfection being common throughout life. Maternal vaccination has emerged as a promising strategy, delivering elevated antibody levels to newborns for immediate protection. However, limited research has explored the protective efficacy of maternal antibodies (matAbs) against secondary RSV infections in offspring. To address this gap, we employed a mouse model of maternal RSV vaccination and secondary infection of offspring to evaluate lung pathology following RSV reinfection in mice with varying levels of maternal antibody (matAb). Additionally, we aimed to investigate the potential causes of exacerbated lung inflammation in offspring with high matAb levels following secondary RSV exposure. Our findings revealed that offspring with elevated levels of maternal pre-F antibody demonstrated effective protection against lung pathology following the initial RSV infection. However, this protection was compromised upon reinfection, manifesting as heightened weight loss, exacerbated lung pathology, increased expression of RSV-A N genes, eosinophilia, enhanced IL-5, IL-13, MUC5AC, and eosinophils Major Basic Protein (MBP) production in lung tissue compared to offspring lacking matAbs. Importantly, these unexpected outcomes were not attributed to antibody-dependent enhancement (ADE) resulting from declining matAb levels over time. Notably, our findings showed a decline in secretory IgA (sIgA), mucosal IgA, and mucosal IgG levels in offspring with high matAb levels post-primary RSV challenge. We propose that this decline may be a critical factor contributing to the ineffective protection observed during secondary RSV exposure. Overall, these findings offer valuable insights into maternal vaccination against RSV, contributing to a comprehensive understanding and mitigation of potential risks associated with maternal RSV vaccination.

## 1 Introduction

Respiratory syncytial virus (RSV) is widely recognized as a common cause of lower respiratory tract infections (LRTI) in children globally, contributing to approximately 33.1 million episodes and 3.2 million hospitalizations annually (1). Furthermore, repeat RSV infections can occur throughout life, even secondary infections within the same year (2–5). The incidence of RSV reinfection in early childhood is about 35% (6). Therefore, RSV infection has a significant disease burden and is considered a global health priority. Achieving vaccination against RSV in early life poses a significant challenge. Severe RSV infections are especially common in infants under 6 months old, a period during which protection heavily depends on matAb. The pre-fusion conformation of the RSV fusion protein (pre-F) is being explored as an antigen in the development of maternal vaccines, due to its capacity to induce high RSV neutralizing antibody titers, which are correlated with a decrease in disease severity (7–11). RSVpreF (Abrysvo) is now approved in the United States as a maternal RSV vaccine to protect infants.

The phase 3 clinical trial demonstrated that maternal immunization with the RSV PreF nanoparticle vaccine effectively transfers matAbs to infants, correlating with a reduced risk of early-onset medically-significant LRTI caused by RSV in infants (10). However, it did not achieve the primary endpoint of reducing medically significant RSV LRTI in infants within 90 days of birth. In an animal model of maternal RSV vaccination, Welliver et al. demonstrated that high titers of neutralizing matAb were associated with the prevention of severe lung disease (12). These studies provided evidences that the high neutralizing matAbs produced by the pre-F maternal vaccine effectively prevent initial RSV infection in both human infants and animal models. Despite the well-established protective effect of matAb against initial RSV infection in infants, little is known about the protective efficacy of matAb against secondary RSV exposure.

Previous studies suggested that initial RSV exposure in neonatal mice predisposes them to more severe airway disease upon reinfection, characterized by significant airway hyperreactivity with marked airway eosinophilia (13, 14). Consequently, if matAb neutralization prevents initial RSV infection in infants and children, does it also mitigate the adverse immune response during subsequent secondary infections? To address this question, we employed a model involving maternal RSV vaccination and secondary RSV exposure in offspring. This allowed us to assess the efficacy and safety of maternal RSV immunization concerning offspring re-exposure to RSV. Furthermore, we investigated the factors contributing to the exacerbation of lung inflammation following secondary RSV infection in the offspring of the high-level matAb group.

## 2 Materials and methods

### 2.1 Mice, immunization, and RSV challenge

Female BALB/c mice were obtained from the Experimental Animals Center of Chongqing Medical University, China. All animal experiments were conducted in compliance with the guidelines established by the Ethics Committee of the Children’s Hospital affiliated with Chongqing Medical University.

Eight-week-old female BALB/c mice were immunized with DS-Cav1 (10 μg per mouse) formulated with Alum (100 μg per mouse) or 100 μl of phosphate-buffered saline (PBS) alone via intramuscular injection before conception and during gestation. Booster immunizations were given at 3-week intervals. The female mice were divided into four groups: three doses of vaccination (3 DS-Cav1), two doses of vaccination (2 DS-Cav1), one dose of vaccination (1 DS-Cav1), and the PBS control group. The final immunization was administered on day 7 of gestation for all groups. No adverse reactions were observed in maternal vaccination. Subsequently, the offspring were exposed to intranasal RSV A2 (5x10^7^ PFU/ml, 30 μl per mouse) or PBS (30 μl per mouse) at postnatal day (PND) 21, and intranasal RSV A2 (5x10^7^ PFU/ml, 100 μl per mouse) at PND 49. The animals were euthanized using isoflurane and cervical dislocation at either 4 or 21 days post-infection (dpi). Animal weights were measured daily following the RSV challenge.

MEDI8897, a recombinant IgG1 human monoclonal antibody derived from the antibody D25, specifically targets the highly conserved epitope ø on pre-F. Varying doses (10 μg/kg, 100 μg/kg, 1 mg/kg, 10 mg/kg) of MEDI8897 were intramuscularly injected into Balb/c mice at 3 weeks of age to simulate matAbs, while the control group received PBS (100 μl). After 24 hours, the mice were exposed to intranasal RSV A2 and subsequently euthanized at either 4 or 21dpi. The viral propagation and quantification procedures followed previously established methods (15).

### 2.2 Antibody analyses, cell counting and cytokine

Infant sera were collected at 1, 3, and 7 weeks postpartum and analyzed using ELISA to measure RSV pre-F specific IgG levels and subtype titers. On day 21 after the initial RSV infection in mice, bronchoalveolar lavage fluid (BAL) was obtained from the right lung via intratracheal instillation of PBS (1.5 ml) and examined for mucosal antibodies (sIgA, IgA, and IgG). BALF cells were collected from mice on day 4 after secondary infection. Differential cell counts to identify eosinophils were performed blindly on Wright-Giemsa-stained BALF cell slides, and at least 100 cells were counted at oil immersion × 1,000 magnification. Supernatants from lung homogenates 4 days after primary and secondary infection were collected to test for cytokine production.

### 2.3 Flow cytometry

The cells derived from Paratracheal lymph nodes tissue were processed using established protocols (16). These cells underwent surface staining with a combination of antibodies, including FVS780 (#565388), IgA-FITC (#553478), IgA-PE (BD Biosciences, #562141), CD45R/B220-PerCp-Cy5.5 (#552771), and CD19-BV421 (#562701) (all antibodies obtained from BD Biosciences unless otherwise specified). Flow cytometric analysis was performed using FlowJo V10.8.1 Software (FlowJo, OR) on a BD FACSCanto instrument.

### 2.4 Histology

At 4dpi, mice were euthanized, and lung tissues were collected for histological assessment. The left lungs were fixed in 10% formalin and subsequently stained with hematoxylin and eosin (H&E) and periodic acid-Schiff (PAS). Following the described methodology (17, 18), inflammation lesions in the HE-stained sections were evaluated using a numeric scale, which includes three scoring components: peribronchial/peribronchiolar inflammation score, perivascular inflammation score, and alveolar inflammation score. Peribronchial/peribronchiolar inflammation was graded as: 0 = no infiltrate, 1 = less than 2 cells thick, 2 = 3 to 5 cells thick, and 3 = more than 5 cells thick. Perivascular inflammation was graded as: 0 = no infiltrate, 1 = less than 4 cells thick, 2 = 5 to 7 cells thick, and 3 = more than 7 cells thick. Lung interstitial spaces inflammation was graded as: 0 = no infiltration, 1 = inflammatory infiltration but no thickening, 2 = significant inflammatory infiltration and mild thickening, and 3 = significant inflammatory infiltration and significant thickening. The mean of these three component scores was then summed up to calculate the total inflammation score. The abundance of PAS-positive mucus-containing cells in each airway was assessed numerically as: 0 as <5% PAS-positive cells, 1 as 5-25%, 2 as 25-50%, 3 as 50-75%, and 4 as >75% (19, 20).

### 2.5 Quantitative real-time PCR

The RNeasy purification kit (Bioflux) was utilized to extract total RNA from the right lung tissue. Subsequently, 1 µg of total RNA was employed to generate cDNA using the QuantiTect Reverse Transcription kit (Accurate Biology). A standard curve was established with the DNA plasmid, and the copy numbers of the RSV-A N gene were determined using the Applied Biosystem 7900HT and subsequently calculated. DNA samples underwent relative quantification, and the resulting relative expression units were normalized to the level of β-actin mRNA. This normalization process yielded a ΔCt value for each sample. Finally, the relative 2^−ΔΔCt^ values were graphically represented. The primers and probes details are shown in **Table 1**.

**Table 1:** Design of nucleic acid reagents used for real-time reverse transcriptase PCR.

Target	Function	Sequence
RSV-A N gene	Forward primer	5′-AGATCAACTTCTGTCATCCAGCAA-3′
	Reverse primer	5′-TTCTGCACATCATAATTAGGAGTATCAAT-3′
	Fluorogenic probe	5′-FAM-CACCATCCAACGGAGCACAGGAGAT-BHQ1-3′
MUC5AC	Forward primer	5′-CAGGGCTGGTACACCTTGTC-3′
	Reverse primer	5′-ACGACATCTGCATCGATTGGA-3′
IL-5	Forward primer	5′-AGAATCAAACTGTCCGTGGGG-3′
	Reverse primer	5′-TCCTCGCCACACTTCTCTTTT-3′
IL-13	Forward primer	5′-AGCATGGTATGGAGTGTGGA-3′
	Reverse primer	5′-TTGCAATTGGAGATGTTGGT-3′
IFN-γ	Forward primer	5′-GGTCAACAACCCACAGGTCC-3′
	Reverse primer	5′-CGAATCAGCAGCGACTCCTT-3′

### 2.6 Statistical analysis

The data were analyzed using GraphPad Prism 9 software, and results were expressed as mean ± standard error of the mean (mean ± SEM). Statistical significance was determined through one-way analysis of variance (ANOVA), with differences considered significant at p < 0.05.

## 3 Results

### 3.1 Antibody decay and protection against initial RSV infection in offspring born to immunized dams

**Figure 1:**
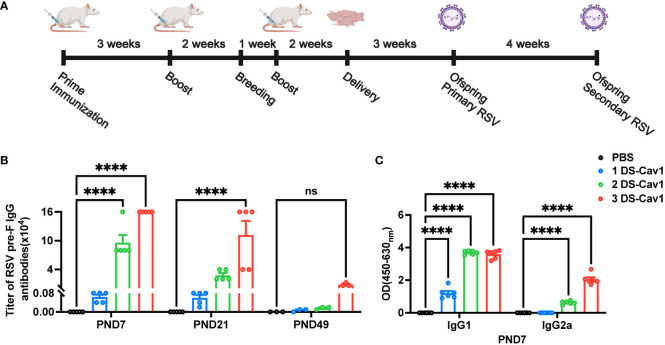
Antibody decay in offspring born to immunized dams. Female BALB/c mice (8 weeks of age) immunized with DS-Cav1 formulated with Alum and PBS vehicle control, with booster immunizations at 3-week intervals. The 3 DS-Cav1 group received immunization at the indicated time **(A)**. All pregnant Balb/c mice groups received a final dose of PBS or preF/Alum vaccine two weeks prior to delivery. Resultant offspring were weaned at PND21, and blood samples were collected at PND7, 21, and 49 to assay for RSV pre-F IgG antibodies **(B)**. Serum collected at PND7 was tested for PreF-specific IgG1 and PreF-specific IgG2a **(C)**. n = 3-6 for each group at each PND age. ****p ≤ 0.0001. ns indicates no significance.

The number of studies assessing the effectiveness of maternal RSV immunization is increasing (10, 12). However, the impact of matAbs on lung inflammation following secondary RSV exposure remains uncertain. Using a maternal immunity model (**Figure 1A**), matAb titers in offspring were evaluated. The analysis of matAb kinetics revealed significantly elevated RSV pre-F IgG levels in offspring born to DS-Cav1-immunized mothers compared to PBS-immunized mothers at all time points, despite a gradual decline over time (**Figure 1B**). The 3 DS-Cav1 group showed higher RSV pre-F IgG titers than the 2 DS-Cav1 and 1 DS-Cav1 groups. At PND7, serum samples from the progeny of DS-Cav1-immunized mothers exhibited a higher concentration of PreF-specific IgG1 compared to PreF-specific IgG2a (**Figure 1C**). These findings indicated placental transmission of maternal RSV pre-F antibodies to offspring, dependent on antibody concentration. Furthermore, maternal antibody levels in offspring serum gradually decreased over time post-birth.

**Figure 2:**
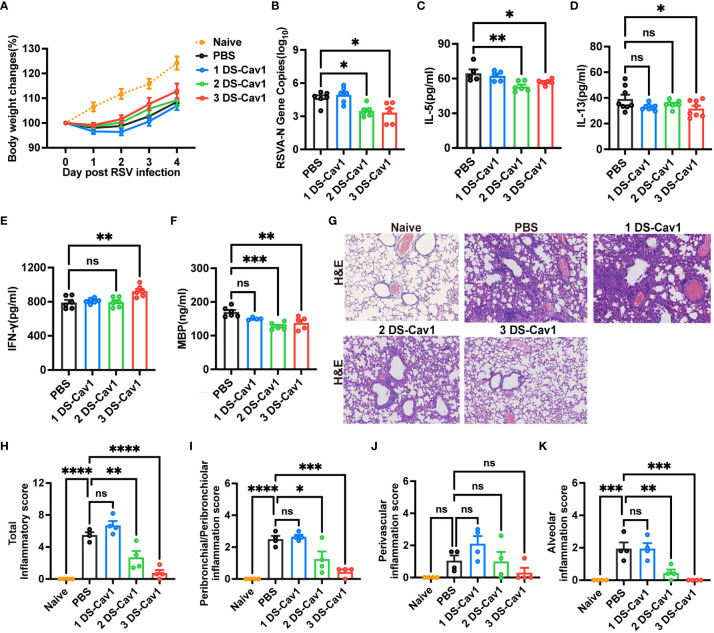
High maternal antibody level protect offspring from initial RSV challenge. At PND21, mice were exposed to 5x10^7^ PFU/ml RSV A2 and weighed daily post-infection **(A)**. At 4 dpi, mice were euthanized, and the right lungs were harvested for the measurement of RSV-A N gene copy numbers using qRT-PCR **(B)**. IL-5 **(C)**, IL-13 **(D)**, IFN-γ **(E)** and MBP **(F)** protein levels in lung homogenates were measured by ELISA. The left lungs were filled with formalin, paraffin embedded, and sectioned for H&E staining. Each panel represents one mouse from the indicated group (scale bar 50 μm) **(G)**. A blinded independent pathologist performed inflammation scoring in all H&E-stained images. As described in Methods, scoring of peribronchial/peribronchiolar inflammation, perivascular inflammation, and alveolar inflammation was broken down in more detail was broken down in more detail **(H-K)**. Data are represented as mean ± SEM (n=4-8 mice per group). *p ≤ 0.05, **p ≤ 0.01, ***p ≤ 0.001, and ****p ≤ 0.0001. ns indicates no significance.

To assess the protective effect conferred by matAb following the initial RSV infection early in life, mice were infected with RSV at PND21. Disease severity was measured by weight loss, with the 3 DS-Cav1 group exhibiting less weight loss compared to PBS controls (**Figure 2A**). At 4dpi, the peak of RSV virus replication in mice, the expression of RSV-A N gene in the lungs was assess using qRT-PCR. Both the 2 DS-Cav1 and 3 DS-Cav1 groups showed a significant reduction in the expression of the RSV-A N gene compared to the PBS group (**Figure 2B**). These findings indicate that elevated levels of matAbs effectively suppressed the expression of the RSV-A N gene in the lungs following initial RSV infection. The concentrations of IL-5, IL-13, and IFN-γ in lung homogenates were measured by ELISA. Protein levels of IL-5 and IL-13 were lower, and IFN-γ levels were higher in the 3 DS-Cav1 group compared to the PBS group. (**Figures 2C–E**). MBP is the major basic protein of eosinophils, and many literatures have shown that it reflects airway hyperresponsiveness and airflow limitation (21–24). In order to indirectly validate airway hyperresponsiveness, MBP protein levels in the lung were measured by ELISA. Following the initial infection, MBP level was significantly lower in the lungs of the 2 DS-Cav1 and 3 DS-Cav1 groups compared to the PBS group (**Figure 2F**). Mice in the PBS group, after RSV challenge, exhibited abundant inflammatory cell infiltration in the peribronchial and perivascular spaces, as well as multifocal alveolitis (**Figure 2G**). This pathology was similar to that observed in mice from the 1 DS-Cav1 group. Notably, mice in the 2 DS-Cav1 and 3 DS-Cav1 groups showed minimal inflammatory pathology in the lungs (**Figure 2G**). The inflammation scores in the 2 DS-Cav1 and 3 DS-Cav1 groups were significantly lower compared to the PBS group (**Figures 2H–K**). These findings suggest that the mice in the 2 DS-Cav1 and 3 DS-Cav1 groups effectively suppressed pneumonia pathology following primary RSV exposure. In conclusion, the offspring in the 3 DS-Cav1 group exhibited elevated matAb titers and superior protection against initial RSV infection compared to the offspring in the PBS group.

### 3.2 Impact of high-titer matAbs on lung pathology during RSV reinfection in offspring

**Figure 3:**
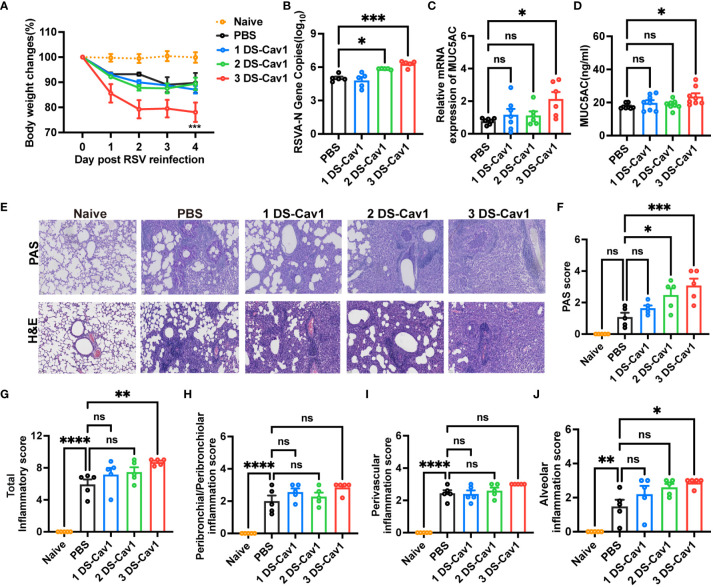
Impact of maternal antibodies on pulmonary pathological damage after RSV reinfection. Mice were exposed to RSV A2 at PND21 and again at PND49. Daily weights were recorded post-infection **(A)**, and samples were collected at 4dpi. RT-PCR was performed to determine RSV N gene copy numbers **(B)** and the relative expression of MUC5AC mRNA **(C)** in the right lung. MUC5AC protein in lung homogenates was measured by ELISA **(D)**. Left lungs were filled with formalin, paraffin-embedded, and sectioned for H&E and PAS staining. Each panel represents one mouse from the indicated group (scale bar 50 μm) **(E)**. All slides were scored by a blinded independent pathologist according to the methods outlined in the study, PAS scores **(F)** and total inflammation scores **(G)** were provided. Inflammation scores around the airways, blood vessels, and interstitial spaces, respectively are shown **(H–J)**. Data are represented as mean ± SEM (n=5-8 mice per group). *p ≤ 0.05, ***p ≤ 0.001, and ****p ≤ 0.0001. ns indicates no significance.

Given the common occurrence of recurrent RSV infections in early childhood (6), it is crucial to evaluate the protective efficacy of matAbs against secondary RSV infections in offspring. Pulmonary lesions were assessed in mice reinfected with the same RSV subtype four weeks post-primary infection. All groups experienced rapid weight loss following secondary RSV infection (**Figure 3A**). Notably, mice in the 3 DS-Cav1 group exhibited a more pronounced degree of weight loss compared to the PBS control group (**Figure 3A**). Furthermore, there was a significant increase in the expression of the RSV-A N gene in the lungs of mice from the 3 DS-Cav1 group after secondary RSV challenge, compared to the PBS group (**Figure 3B**). These findings suggest that mice born with elevated matAb levels demonstrated an increased RSV burden in the lungs upon secondary exposure. The relative mRNA expression and protein levels of MUC5AC were significantly higher in the lungs of the 3 DS-Cav1 group compared to the PBS group (**Figures 3C, D**). Histological studies of lung samples were performed to quantify inflammation scores using H&E staining and to assess mucin hypersecretion using PAS staining (**Figure 3E**). Compared to the PBS group, the 2DS-CAV1 and 3 DS-Cav1 groups had significantly higher PAS scores following secondary infection (**Figure 3F**). Additionally, enhanced lung inflammation was observed in the 3 DS-Cav1 group compared to the PBS group. Total inflammation scores were markedly higher in the 3 DS-Cav1 group than in the PBS group, while the scores for the 1 DS-Cav1 and 2 DS-Cav1 groups resembled those of the PBS group (**Figure 3G**). Following secondary RSV challenge, inflammation scores within the lung interstitial spaces were significantly elevated in the 3 DS-Cav1 group compared to the PBS group (**Figure 3J**), with no notable differences observed in scores related to airways or blood vessels (**Figures 3H, I**). Overall, these results suggest that the 3 DS-Cav1 mice exhibited exacerbated pneumonitis pathology upon secondary RSV challenge.

**Figure 4:**
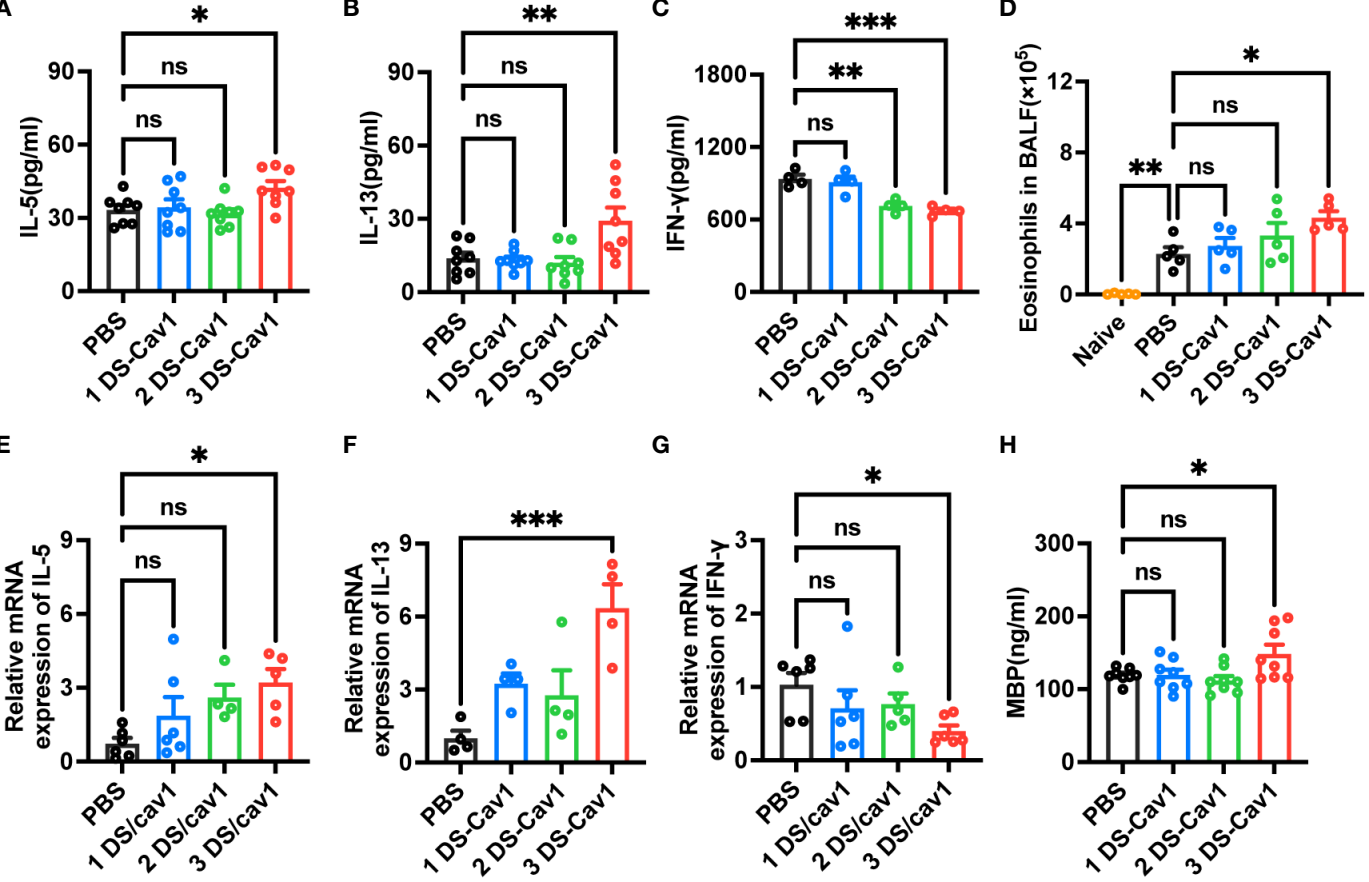
Impact of maternal antibodies on inflammatory factors and eosinophils in lungs after RSV reinfection. Samples were collected at 4dpi after reinfection in mice. IL-5 **(A, E)**, IL-13 **(B, F)**, and IFN-γ **(C, G)** mRNA and protein levels in the lungs were detected by RT-PCR and ELISA. Eosinophils in BALF were counted. **(D)**. Protein levels of MBP was detected in lung homogenates by ELISA **(H)**. Data are represented as mean ± SEM (n=4-8 mice per group). **p ≤ 0.01, and ***p ≤ 0.001. ns indicates no significance.

Numerous studies have shown that primary RSV exposure in infancy leads to exaggerated Th2 responses upon RSV reinfection (13, 14, 25). To investigate whether the presence of matAbs during initial infant RSV exposure affects subsequent Th2 bias upon RSV re-exposure, the mRNA and protein levels of IL-5, IL-13 and IFN-γ in lung tissues were determined by ELISA and RT-PCR on day 4 after secondary RSV infection. Notably, compared with the PBS offspring, the 3 DS-Cav1 group lungs increased mRNA and protein concentrations of Th2 cytokines, IL-13 and IL-5, and decreased mRNA and protein concentrations of Th1 cytokines, IFN-γ (**Figures 4A–C, E–G**). The 2DS-CAV1 group also had significantly reduced IFN-γ at the protein level (**Figure 4C**). The number of eosinophils in BALF was significantly increased in the 3 DS-Cav1 group compared to the PBS group (**Figure 4D**). Additionally, following secondary infections, MBP levels were significantly elevated in the lung tissue of the 3 DS-Cav1 group (**Figure 4H**). Our findings indicate that the neutralizing matAbs during the initial infant RSV exposure fails to mitigate the establishment of a Th2-skewed response upon RSV rechallenge. In summary, these data underscore the pronounced Th2-skewed response, pulmonary eosinophilia, and increased MBP in offspring from the 3 DS-Cav1 group compared to the PBS group following secondary RSV exposure.

### 3.3 Impact of maternal antibody decay on lung pathology

**Figure 5:**
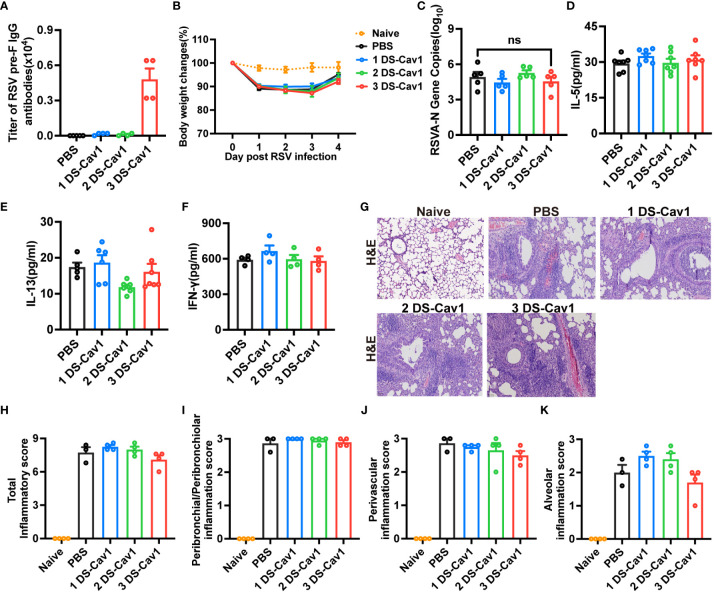
MatAbs that weakened over time no longer provided significant protection and did not lead to increased pathology. Mice were initially exposed to PBS at PND21 and subsequently to RSV A2 at PND49. At PND49, blood samples were collected before infection and tested for RSV pre-f IgG antibody **(A)**. Daily weights were recorded following RSV infection **(B)**, and samples were collected at 4dpi. RT-PCR was performed to determine RSV N gene copy numbers **(C)** in the right lung. IL-5 **(D)**, IL-13 **(E)**, and IFN-γ **(F)** protein levels in lung homogenates were detected by ELISA. At 4dpi, the lung tissues were collected and stained with H&E. Representative photographic images of lung histopathology **(E)** were captured. Scale bars represent 50 μm. Inflammation scores were separately assessed at the peribronchial/peribronchiolar, perivascular, and interstitial sites and summarized **(G–K)**. Data are expressed as mean ± SEM (n=3-7). ns indicates no significance.

While high levels of matAbs provided protection against primary RSV infection at PND21, reinfection at PND49 resulted in heightened lung inflammation compared to mice without matAbs. To investigate whether this increased pathology is caused by the decay of high matAb levels over time, mice received intranasal injections of PBS at PND21 and RSV at PND49. Blood samples were collected prior to PND49 infection and tested for attenuated maternal RSV pre-f IgG antibody titers (**Figure 5A**), which had markedly attenuated to quite low levels in the 3 DS-Cav1 group. The antibody titers in the 1 DS-Cav1 and 2 DS-Cav1 groups were significantly attenuated and close to those of the PBS group. Weight loss was similar in all groups (**Figure 5B**). RSV-A N gene expression in the lungs of mice from the 3 DS-Cav1 group was not elevated compared to the PBS group (**Figure 5C**). There were no significant differences in IL-5, IL-13 and IFN-γ protein levels in all groups. (**Figures 5D–F**). Lung pathology and inflammation scores in the 3 DS-Cav1 group were not significantly different from those in the PBS group (**Figures 5G–K**). Likewise, the 1 DS-Cav1 and 2 DS-Cav1 groups did not exhibit enhanced lung pathology. These results suggest that matAbs, which weaken over time, do not exacerbate lung pathology.

### 3.4 Reduced secretion of sIgA, mucosal IgA and mucosal IgG after primary RSV infection in offspring with high titer antibodies

Previous studies have highlighted the superior protective role of mucosal IgA in RSV infection compared to serum antibodies (26, 27). Additionally, mucosal IgG levels exhibit a stronger correlation with RSV load and inflammation than plasma IgG levels (28). Therefore, we hypothesized that exacerbated lung inflammation upon reinfection in the 3 DS-Cav1 group might be linked to respiratory mucosal antibody responses. To explore how matAbs alter the respiratory mucosal immune response following initial RSV infection in offspring, mice were infected at PND21, and antibodies in BAL were assessed.

**Figure 6:**
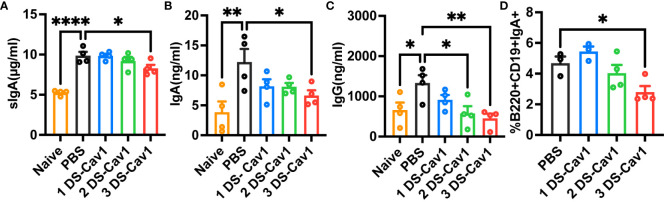
High titer matAbs suppressed the mucosal antibody responses in the offspring. The offspring were exposed to RSV at PND21. BAL was collected at 21dpi and detected by ELISA for sIgA, IgA, and IgG **(A–C)**. Paratracheal lymph nodes were collected, and B cells expressing surface IgA were measured by flow cytometry **(D)**. Data are expressed as mean ± SEM (n=3-4). *p ≤ 0.05, **p ≤ 0.01, and ****p ≤ 0.0001.

In mice with initial RSV exposure at PND21, levels of sIgA, IgA, and IgG were significantly lower in BAL of the 3 DS-Cav1 group compared to the PBS group (**Figures 6A–C**), with no significant difference observed between mice infected at the 1 DS-Cav1 and the PBS group. The proportion of IgA-expressing B cells at 21dpi was significantly lower in the 3 DS-Cav1 group mice compared to the PBS group (**Figure 6D**). The findings indicate that high titer matAbs inhibit the secretion of sIgA, mucosal IgA, and mucosal IgG in the offspring subsequent to the initial RSV infection.

### 3.5 Inhibition of mucosal antibody secretion by pre-existing high levels of pre-F antibody

**Figure 7:**
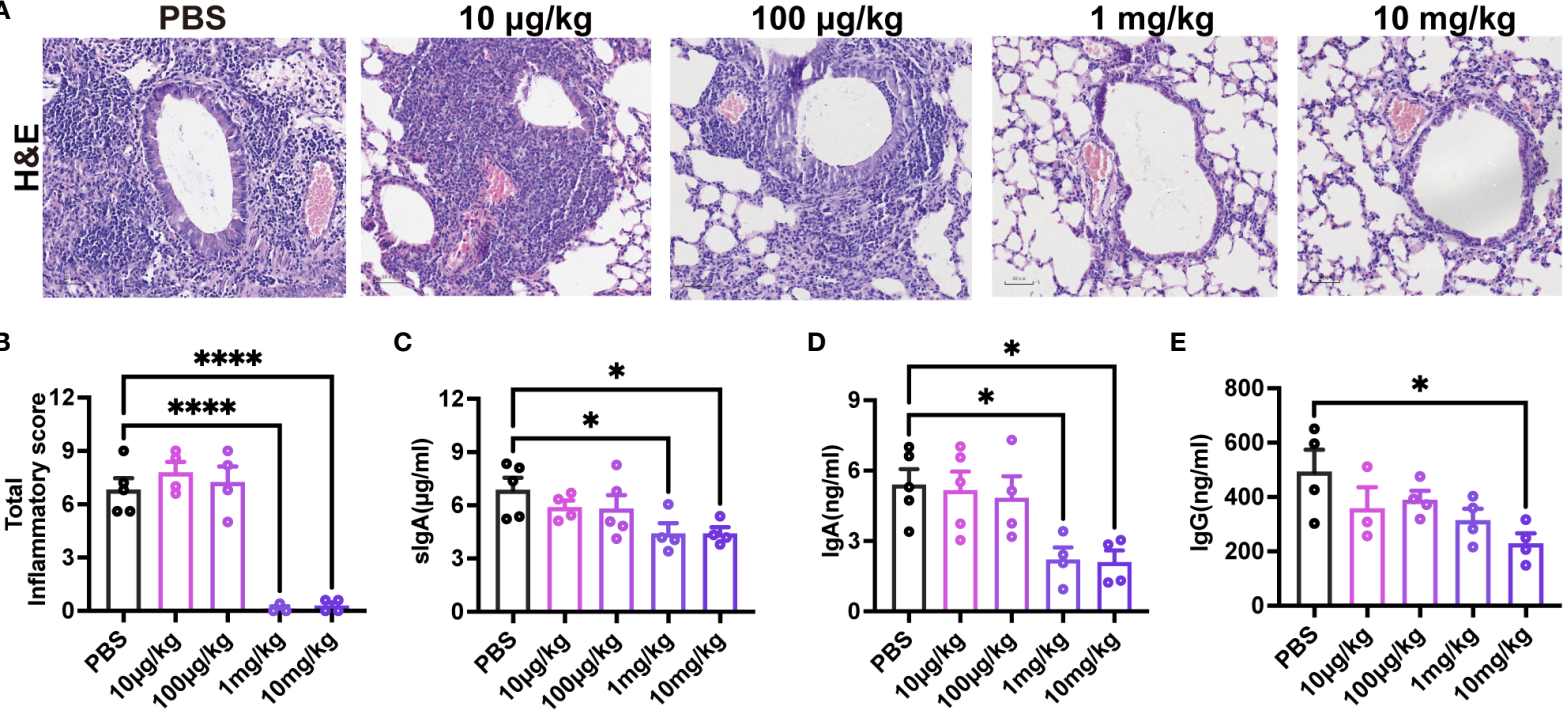
High levels of pre-existing MEDI8897 inhibit mucosal antibody responses. Lung tissues were collected from mice on day 4 post-RSV infection for H&E staining, and representative photographic images illustrating histopathology in the lung are presented **(A)**. Scale bars represent 50 μm. Inflammation scores of the left lung on day 4 post-infection are depicted **(B)**. BAL was collected at 21dpi and analyzed using ELISA for sIgA, IgA, and IgG **(C-E)**. Data are expressed as mean ± SEM (n=3-5). *p ≤ 0.05 and ****p ≤ 0.0001.

To assess the impact of pre-existing pre-F antibodies on respiratory mucosal immunity following RSV infection, 3-week-old mice received varying doses of MEDI8897. Evaluation of lung pathology and inflammation scores (**Figures 7A, B**) indicated that the 1 mg/kg and 10 mg/kg groups effectively reduced RSV infection. As anticipated, mice with high levels of MEDI8897 (10 mg/kg) exhibited significantly lower levels of sIgA, IgA, and IgG in BAL after initial RSV exposure compared to the PBS group (**Figures 7C–E**). These findings suggest that pre-existing high levels of pre-F antibodies inhibit the secretion of sIgA, mucosal IgA, and mucosal IgG following RSV infection.

## 4 Discussion

Maternal immunization not only avoids the risk of exacerbating the condition of young infants with early-life RSV vaccination but also protects them from severe RSV infection in the months following birth (29). Studies on human serum post-natural infection have revealed that the most potent neutralizing antibodies target RSV PreF protein (30, 31), suggesting that endowing infants with high levels of anti-RSV PreF antibodies through maternal immunization could effectively prevent severe RSV infection in this vulnerable population. While maternal RSV vaccination has demonstrated efficacy in reducing medically significant RSV lower respiratory tract infections in infancy (10), the emphasis has primarily been on the initial RSV infection in offspring. However, reinfection in infants and young children, particularly in those with chronic lung or heart conditions, is common. Few studies have explored the impact of matAbs on offspring immunity and lung histology during secondary RSV infection or on mucosal immunity during primary RSV infection.

Our findings indicate that elevated levels of maternal preF IgG antibodies provide protection to offspring against primary RSV challenge. However, these antibodies also lead to increased lung inflammation and a skewed Th2 immune response following RSV reinfection. Crucially, the decline in matAbs over time did not correlate with heightened pathology. These findings indicate that high levels of matAbs might alter the respiratory mucosal immune response in offspring, potentially exacerbating lung inflammation during secondary RSV infections. This underscores the importance of establishing robust mucosal immunity against subsequent RSV reinfection.

Elevated levels of matAbs confer a protective immunity against primary RSV infection early in life. However, lung inflammation during reinfection is more severe compared to mice lacking matAbs. We hypothesize two possible explanations for this unexpected finding.

Firstly, known risk factors for ADE include low antibody affinity, limited neutralizing ability, and low antibody quantity (concentration). During RSV reinfection, matAbs gradually decrease from high to low titers over time. Immunization against the DS-Cav1 protein elicits antibodies targeting six epitopes, with those directed against antigenic site ø exhibiting higher affinity and neutralizing ability than those directed against other epitopes (32). The presence of those lower-affinity, lower-neutralizing antibodies may increase the risk for ADE. To assess whether the decline in maternal RSV pre-F specific IgG levels triggers ADE, leading to excessive Th2 inflammatory responses and exacerbated lung damage in mice, offspring received PBS at 3 weeks of age and intranasal RSV at 7 weeks of age. Our findings indicate that the diminishing matAbs no longer confer protection and do not exacerbate lung lesions. This suggests that matAb attenuation in offspring does not induce ADE in our model, underscoring the significant impact of the immune response to primary RSV infection on reinfection.

Secondly, prior researches have demonstrated that viral infections may trigger persistent germinal center responses and antibody production within the lungs (33, 34). RSV primarily infects the body by infiltrating the epithelial cells of the respiratory mucosa, making the respiratory mucosa the primary defense against RSV infection. Respiratory mucosal immunity is primarily mediated by IgA, particularly sIgA, which plays a major role in fighting against viral invasion (35). Despite their lower abundance compared to serum antibodies, mucosal IgA exhibits superior protective efficacy as they directly neutralize the virus without eliciting complement activation-induced inflammatory responses (27). In infants infected with RSV, elevated levels of mucosal IgG were more closely correlated with reduced RSV viral load compared to plasma IgG levels (28). Our research revealed that heightened maternal antibody levels hindered the production of sIgA, mucosal IgA, and mucosal IgG following the initial RSV infection in offspring. Therefore, the diminished levels of sIgA, mucosal IgA, and mucosal IgG may partially account for the heightened lung inflammation observed during reinfection.

Our findings indicate that only elevated levels of matAbs contribute to heightened lung inflammation following secondary RSV infection. A study conducted within the Danish National Birth Cohort concluded that while maternally transmitted RSV neutralizing antibodies assist in safeguarding infants from RSV-related hospitalization, high levels of these antibodies may increase the risk of recurrent wheezing (36). This observation aligns with our results, suggesting that high levels of RSV matAb may potentially pose a risk. Therefore, careful consideration is warranted regarding the quantity of RSV-specific IgG matAb transferred to offspring. Additionally, our results reveal that offspring from mothers immunized with the preF/Alum vaccine exhibit higher levels of IgG1 compared to IgG2a. Future investigations could explore different vaccine adjuvants to modulate the IgG subclasses produced, thereby elucidating whether enhancing IgG2a transfer improves the outcome of RSV reinfection. In addition, the stability of DS-Cav1 did not meet expectations during the stability evaluation of recombinant proteins. Subsequent studies should investigate whether the outcome of secondary RSV infection in offspring can be improved when the mother is vaccinated with other more stable pre-F antigen vaccines.

Our data do not contradict the prevailing understanding that matAbs offer immediate protection against RSV infection. However, our study underscores the significant impact of matAbs on mucosal immunity and secondary RSV infection in offspring. We suggest that, in addition to current maternal vaccine strategies, infants, especially those prone to recurrent infection following initial exposure, may benefit from strategies aimed at enhancing mucosal antibodies to strengthen immunity and prevent severe disease in subsequent reinfections. MatAb titers alone are inadequate for predicting the protective and pathological outcomes of recurrent RSV infections in offspring. Therefore, determining the optimal quantity and quality of matAbs transferred to offspring is essential for understanding and mitigating potential risks associated with maternal RSV vaccines.

## Data availability statement

The raw data supporting the conclusions of this article will be made available by the authors, without undue reservation.

## Ethics statement

The animal study was approved by The Ethics Committee of the Children’s Hospital affiliated with Chongqing Medical University. The study was conducted in accordance with the local legislation and institutional requirements.

## Author contributions

JM: Writing – original draft, Writing – review & editing, Conceptualization, Data curation, Formal analysis, Investigation, Methodology, Project administration, Resources, Software, Supervision, Validation, Visualization. TG: Writing – review & editing. TL: Formal analysis, Investigation, Methodology, Writing – review & editing. SL: Formal analysis, Methodology, Writing – review & editing. LZ: Formal analysis, Writing – review & editing. XZ: Formal analysis, Writing – review & editing. CM: Formal analysis, Writing – review & editing. HB: Formal analysis, Writing – review & editing. ZJ: Methodology, Resources, Writing – review & editing. XK: Methodology, Resources, Writing – review & editing. XW: Methodology, Resources, Writing – review & editing. ZF: Formal analysis, Project administration, Supervision, Writing – review & editing. DT: Conceptualization, Formal analysis, Funding acquisition, Project administration, Resources, Supervision, Validation, Visualization, Writing – review & editing.
